# Capturing Differences in the Regulation of LRRK2 Dynamics and Conformational States by Small Molecule Kinase Inhibitors

**DOI:** 10.1021/acschembio.2c00868

**Published:** 2023-04-12

**Authors:** Jui-Hung Weng, Wen Ma, Jian Wu, Pallavi Kaila Sharma, Steve Silletti, J. Andrew McCammon, Susan Taylor

**Affiliations:** † Department of Pharmacology, University of California, San Diego, California 92093, United States; ‡ Department of Chemistry and Biochemistry, University of California, San Diego, California 92093, United States

## Abstract

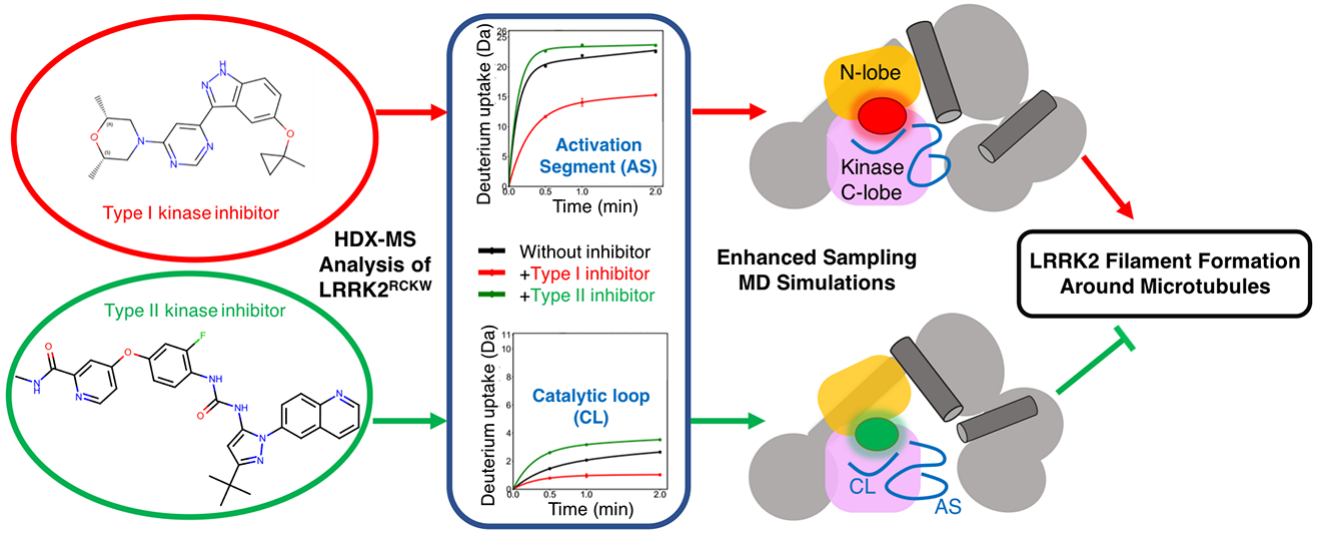

Mutations in the human leucine rich repeat protein kinase-2 (LRRK2) create risk factors for Parkinson’s disease, and pathological functions of LRRK2 are often correlated with aberrant kinase activity. Past research has focused on developing selective LRRK2 kinase inhibitors. In this study, we combined enhanced sampling simulations with HDX-MS to characterize the inhibitor-induced dynamic changes and the allosteric communications within the C-terminal domains of LRRK2, LRRK2^RCKW^. We find that the binding of MLi-2 (a type I kinase inhibitor) stabilizes a closed kinase conformation and reduces the global dynamics of LRRK2^RCKW^, leading to a more compact LRRK2^RCKW^ structure. In contrast, the binding of Rebastinib (a type II kinase inhibitor) stabilizes an open kinase conformation, which promotes a more extended LRRK2^RCKW^ structure. By probing the distinct effects of the type I and type II inhibitors, key interdomain interactions are found to regulate the communication between the kinase domain and the GTPase domain. The intermediate states revealed in our simulations facilitate the efforts toward *in silico* design of allosteric modulators that control LRRK2 conformations and potentially mediate the oligomeric states of LRRK2 and its interactions with other proteins.

## Introduction

**Figure 1:**
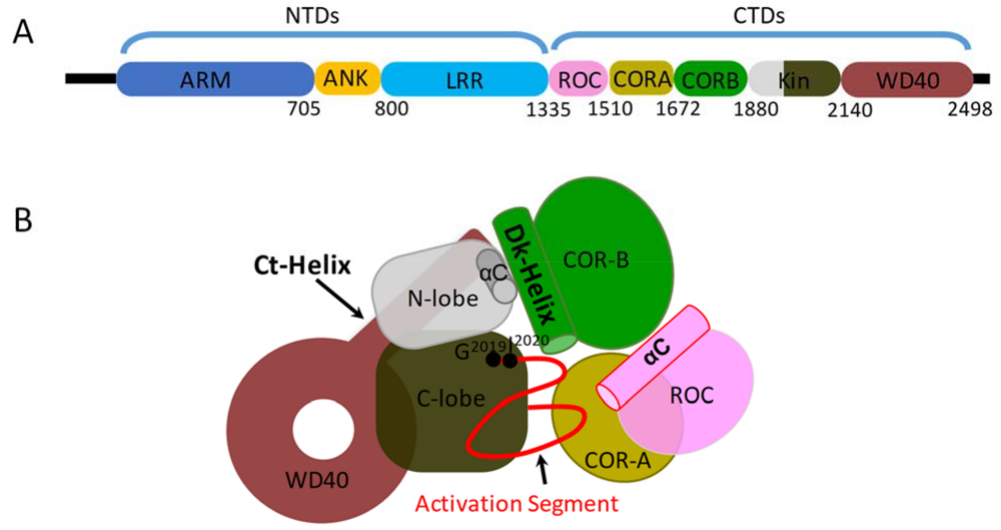
Schematic domain organization of LRRK2. (A) Full length LRRK2 consists of the armadillo domain (ARM); ankryn repeat (ANK); leucine-rich repeat (LRR); Ras of complex (ROC), GTPase domain; C-terminal of Roc domain (COR); kinase domain; and WD40 domain. The N-terminal domains (NTDs) contain the ARM, ANK, and LRR domains, and the C-terminal domains (CTDs) contain the ROC, COR, kinase, and WD40 domains. (B) The model of LRRK2 CTDs from the Cryo-EM structure shows how the kinase domain is surrounded by the flanking domains.

LRRK2 (leucine rich repeat protein kinase-2) is a large 2527 residue multidomain protein that contains armadillo (ARM), ankyrin (ANK), and leucine-rich (LRR) repeats followed by a tandem Roco type GTPase consisting of a ROC and COR domain, a Ser/Thr kinase (KIN) domain, and a C-terminal WD40 domain (Figure 1A). Mutations in LRRK2 cause it to become a risk factor for Parkinson’s disease (PD), and the pathological functions of LRRK2 correlate mainly with aberrant kinase activity.^1−6^ The modulation of LRRK2 kinase activity via the design of small-molecule inhibitors has thus been a central focus for treating PD,^7−10^ and most of the studies so far have focused on kinase inhibitors.

There are two widely studied classes of kinase inhibitors, ATP-competitive type-I inhibitors that bind to the kinase domain and lock it into a closed and active-like conformation and type-II inhibitors that can be either ATP-competitive or noncompetitive with ATP and typically maintain the kinase in an open inactive conformation.^11^ To date, most drug research focuses on type I inhibitors because of their better selectivity,^12,13^ and many experiments have been conducted to increase their efficacy. Structural studies based on homology models or a kinase domain surrogate were used for designing optimal inhibitors with better potency and exquisite selectivity.^14,15^ However, some potent type I kinase inhibitors, such as MLi-2 (Merck LRRK2 Inhibitor 2), appear to stabilize a disease-like cellular phenotype where LRRK2 accumulates on microtubules (MT).^16^ In contrast, some high affinity type II kinase inhibitors, such as Rebastinib, Ponatinib, and GZD-824, appear to lock LRRK2 into a conformation that is unable to bind to MT.^17,18^ Both type I and type II LRRK2 inhibitors inhibit LRRK2-mediated phosphorylation of Rab proteins, and both can also stimulate mitophagy, which is negatively regulated by LRRK2.^19^ Only type I inhibitors, however, reduce the phosphorylation of well-studied LRRK2 biomarker sites at the N-terminal region of LRRK2 by inducing dephosphorylation while type II inhibitors do not.^20^ This suggests that, in addition to the intrinsic kinase activity toward Rab substrates, the conformation of the LRRK2 protein, likely regulated by the opening and closing of the kinase domain and modulated by binding of 14–3–3 proteins,^21,22^ plays an important role in mediating the steady state phosphorylation of the biomarker sites, which regulate LRRK2 function.^20^

To date, several high-resolution LRRK2 structures are available of both full length LRRK2 and a truncated version of LRRK2 that contains only the C-terminal domains (LRRK2^RCKW^), and these provide an incredibly valuable resource for delving more deeply into the mechanistic features that regulate LRRK2 structure and function.^17,23^ The kinase domain is surrounded by the CORA, CORB, and WD40 domains (Figure 1B), and LRRK2 is one of the only kinases that has a GTPase domain embedded in the same polypeptide. With LRRK2^RCKW^ we can thus capture the direct cross talk between these two major signaling motifs that control so much of biology. Both active and inactive LRRK2 structures have been captured by cryo-EM, and these structures reveal distinct domain movements that resemble the closed and open states of the breathing dynamics.^24^ However, no structure of the LRRK2 kinase domain in complex with small-molecule inhibitors has been reported. Other studies on the binding mode between LRRK2 and kinase inhibitors have been mostly based on molecular docking calculations or structures from homologue models of the LRRK2 kinase domain or LRRK2-like mutation studies, and they all focused on the kinase domain alone.^15^ A kinase inhibitor, DCLK1-IN-1, induces conformational changes in DCLK1’s kinase domain but not its microtubule-associated protein (MAP) function; the relationship between the kinase activity and its MAP function is not well understood.^25,26^ A key question is what roles the conformation of the kinase domain plays in the intrinsic regulatory processes that mediate subcellular location and activation of LRRK2.^27^

In this study, using Gaussian accelerated molecular dynamics MD (GaMD) simulations coupled with hydrogen–deuterium exchange mass spectrometry (HDX-MS), we studied the C-terminal domains of LRRK2, which include the ROC, COR, kinase, and WD40 domains (LRRK2^RCKW^), to show how LRRK2 dynamics is affected differently by binding to type I vs type II kinase inhibitors. The results show how the N and C lobes function as independent rigid bodies that are stabilized by MLi-2 in a closed and active-like conformation but stabilized by Rebastinib in an open conformation where the two domains are uncoupled. The critical regulatory triad (K1906 in β3, E1920 in the αC helix, and D2017 in the DYG motif) formed in the MLi-2 structure allows the regulatory spine (R-spine) to assemble in an active-like conformation. In contrast, the triad is broken in the Rebastinib-bound LRRK2^RCKW^ structure where D2017 is far from the K1906-E1920 ion pair, and in this structure the R spine is broken. Our studies also validate the importance of the dynamic features of the Dk helix, which plays a crucial role in bridging the two catalytic domains, the kinase and GTPase domains.^28^

The HDX-MS data reveal that, unlike the type I inhibitor, MLi-2, which reduces the deuterium uptake of the entire kinase domain and the flanking domains that lie in close proximity to the kinase domain, the type II inhibitor, Rebastinib, reduces the local deuterium uptake in the N lobe but actually increases deuterium uptake in the C lobe of the kinase domain. Using GaMD simulations, we demonstrate that the type I inhibitor stabilizes the closed, active-like conformation of the kinase domain and promotes the compact domain orientation of LRRK2^RCKW^. In contrast, the type II inhibitor locks the kinase domain into an open conformation by separating the N and C lobes, which in turn stabilizes the domains of LRRK2^RCKW^ in an extended conformation. The dynamic changes in the kinase domain that propagate through the Dk helix also lead to different conformations of the ROC domain, which potentially affect the GTPase activity. The dynamic and conformational changes described here may also participate in mediating the scaffold and oligomerization properties of LRRK2 in signaling, which leads to different LRRK2 functions.

## Results

### HDX-MS Analysis Shows Different Effects of Type I and Type II Kinase Inhibitors on LRRK2

**Figure 2:**
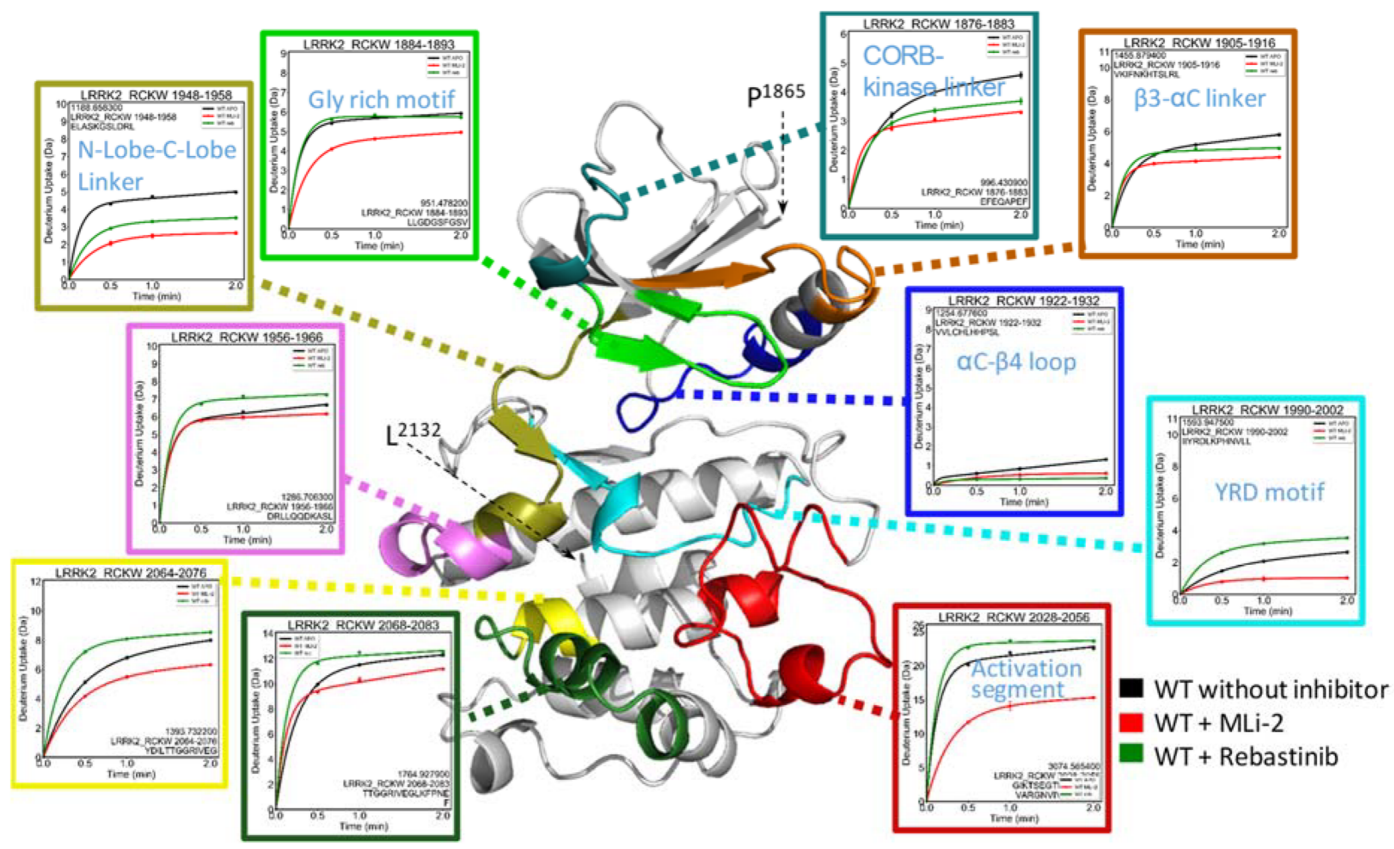
The deuterium uptake of the LRRK2 kinase domain. The deuterium uptakes of the selected peptides are plotted and mapped on the kinase model (residue 1865–2132). For all peptides, the uptake is reduced in the presence of Mli-2. Binding of Rebastinib reduces the uptake of CORB–kinase linker, β3−αC linker, N-Lobe–C-Lobe linker, but the effect is less than that of MLi-2. In contrast, the uptake increases for peptides that cover the C lobe of the kinase when binding to the Rebastinib.

To gain insight into how type I and type II inhibitors affect the solvent accessibility of the catalytic domains of LRRK2, we used HDX-MS to measure the deuterium uptake changes of LRRK2^RCKW^ in the presence of MLi-2 and Rebastinib. Peptides with matching residues across all conditions were combined, covering 89.9% of the total protein (Figure S1). We then mapped the HDX-MS data onto the LRRK2^RCKW^ model, which is based on the LRRK2^RCKW^ structure (PDB: 6vno),^17^ and focused on the kinase domain first (Figure 2 and Table S1). We found that while MLi-2 reduces the deuterium uptake of the kinase domain, Rebastinib shows a different effect on the N lobe and C lobe of the kinase domain. For peptides that cover the CORB–kinase linker (aa 1876–1883), β3−αC linker (aa 1905–1916), and N-Lobe–C-Lobe linker (aa 1948–1958), binding of Rebastinib reduces the H–D exchange but not as effectively as MLi-2. For example, in the linker peptide (aa 1948–1958), Rebastinib reduces the deuterium uptake at 2 min from 50% to 35% of its maximum uptake (5 to 3.5 Da out of maximum 10 Da), while MLi-2 reduces the uptake to 27% (2.7 Da). In contrast, for the glycine-rich loop (aa 1884–1893), which is highly protected by MLi-2, the deuterium uptake was unchanged when binding to Rebastinib. For the αC−β4 loop, the low deuterium uptake indicated that it is almost completely shielded from the solvent in the condition without an inhibitor, and both MLi-2 and Rebastinib reduce their deuterium uptake even more.

Interestingly, binding of Rebastinib does not reduce the deuterium uptake in the C lobe of the kinase domain. Instead, the deuterium uptake is actually increased in several regions in the C lobe (Figure 2). The activation segment is highly flexible and has high deuterium uptake. The peptide that covered this region (aa 2028–2056) has a higher deuterium uptake when binding to Rebastinib, in contrast to the effect of MLi-2, which has a significant reduction in deuterium uptake. The catalytic loop (aa 1990–2002), including the YRD motif, also becomes more solvent exposed (without inhibitor, 24% vs Rebastinib bound, 32% of the maximum uptake at 2 min) when binding to Rebastinib, whereas binding of MLi-2 reduced its deuterium uptake to 9.3% at 2 min. Peptides at the C-terminal end of αF (aa 2064–2076), the αF−αG loop (aa 2068–2083), and the loop between the hinge region and αE (aa 1956–1966) all become more solvent exposed when binding to Rebastinib. Unlike MLi-2 that reduces the deuterium uptake of the entire kinase domain, binding of Rebastinib only protects a localized portion of the N lobe of the kinase domain from solvent while the C lobe becomes globally more solvent exposed when binding to Rebastinib.

#### Binding of Kinase Inhibitors Changes the Conformation of LRRK2

**Figure 3:**
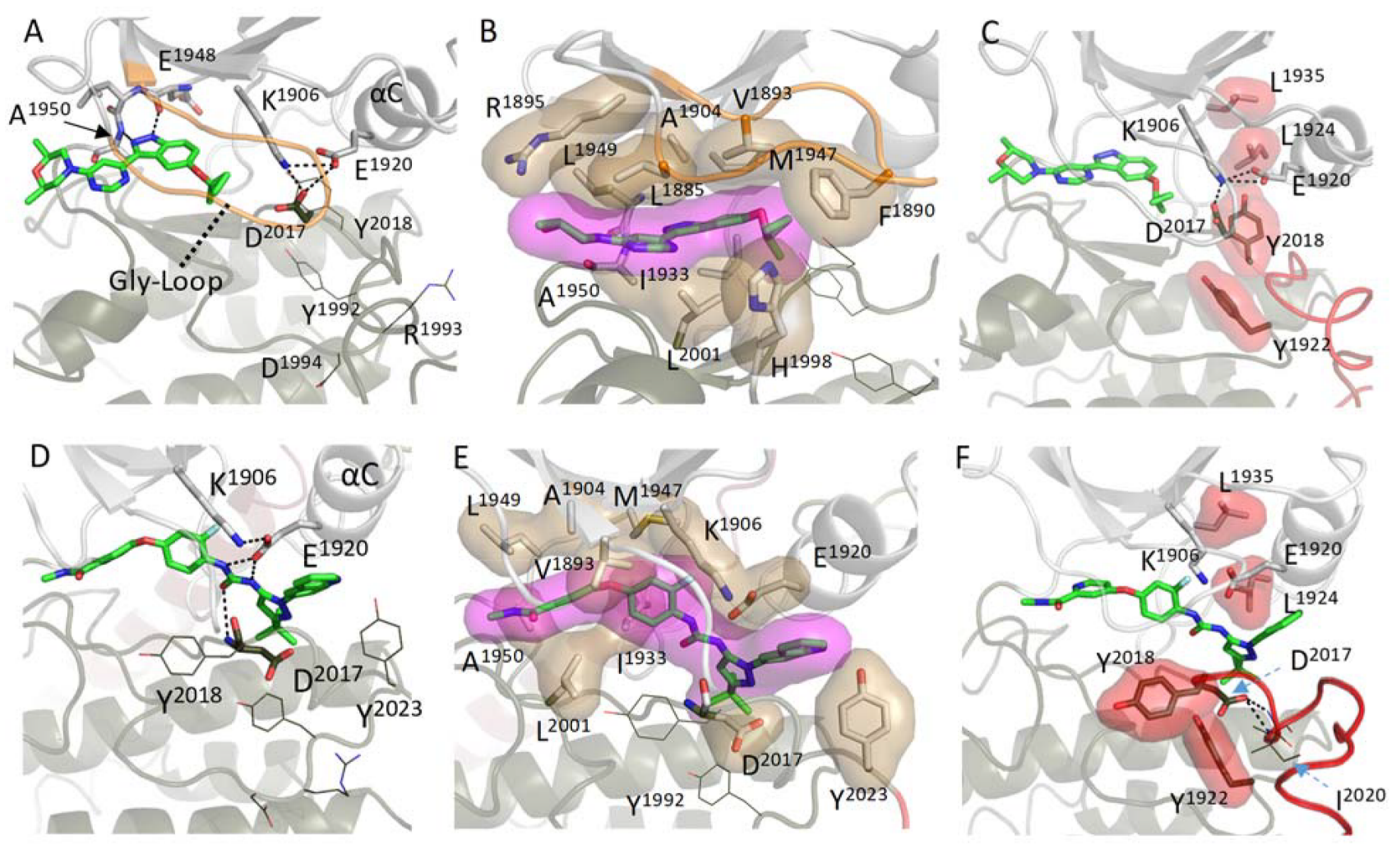
Structure of the binding pocket of the LRRK2 kinase domain with inhibitors. (A) MLi-2 binds in the ATP binding pocket of the kinase domain and forms hydrogen bonds with residues R1895 and E1948. (B) Hydrophobic interactions occuring between MLi-2 and residues in the Gly-rich loops and the hinge region are highlighted. (C) The MLi-2 bound LRRK2 kinase samples the active-like conformation where the regulatory triad (K1906, E1920, and D2017) and regulatory spine (L1935, L1924, Y2018, and Y1922) are assembled. (D) Rebastinib displaces the Tyr of the DYG-motif and binds to residues E1920 and D2017. In addition, binding of Rebastinib prevents the αC helix from moving toward the C lobe. (E) Hydrophobic interactions with Rebastinib create a wedge between the N and C lobes. (F) Rebastinib blocks the assembly of R-spine, and the kinase domain is locked in the DYG-out, inactive conformation.

To explore the allosteric impact of inhibitor binding, we carried out three GaMD simulations: LRRK2^RCKW^ (RCKW), LRRK2^RCKW^ in complex with MLi-2 (RCKW/MLi-2), and LRRK2^RCKW^ with Rebastinib (RCKW/Rebastinib). The RCKW structure was built based on the reported cryo-EM structure (PDB: 6VNO), and the inhibitor coordinates were obtained by superimposing the reported respective inhibitor-bound kinase structures through the conserved αE and αF helices (PDB: 5OPB for MLi-2 and PDB: 6MWE for Rebastinib).^14,29^ The RCKW/inhibitor models were relaxed using energy minimization, applied first to the side chain and then to the overall structure. Ten replicated simulations were carried out for each of the models (RCKW, RCKW/MLi-2, and RCKW/Rebastinib). Both inhibitors stayed in the binding pocket throughout the simulation, and the RMSD value of the inhibitors indicated that both inhibitors bound to the LRRK2 protein stably. To show the detailed binding modes of the inhibitors, the representative structure of RCKW/inhibitor is shown in Figure 3. MLi-2, which is an ATP analog, occupied the ATP binding pocket of the LRRK2 kinase domain and was capped by the Gly-rich loop (aa 1886–1893; Figure 3A). The morpholine group in MLi-2 was surrounded by residues in the hinge/linker region (aa 1949–1954) and R1895 on the β2 strand. L2001 and L1985, two C-spine residues, clamped to MLi-2 (Figure 3B) and E1948, at the end of β5, formed a stable H-bond with MLi-2. Two hydrophobic residues in particular, F1890 in the G-Loop and L2001 and F2003 of the C lobe fuse the N and C lobes together. A1950 also interacted with MLi-2 through a H bond to its backbone in some of the frames. Binding of MLi-2 stabilized the DYG-in/BLBplus conformation.^30^ The LRRK2 kinase was in a “DYG-In” conformation for 90% of the simulation time, which indicates that binding of MLi-2 stabilizes the LRRK2 kinase in an active-like state (Figure S2).

**Figure 4:**
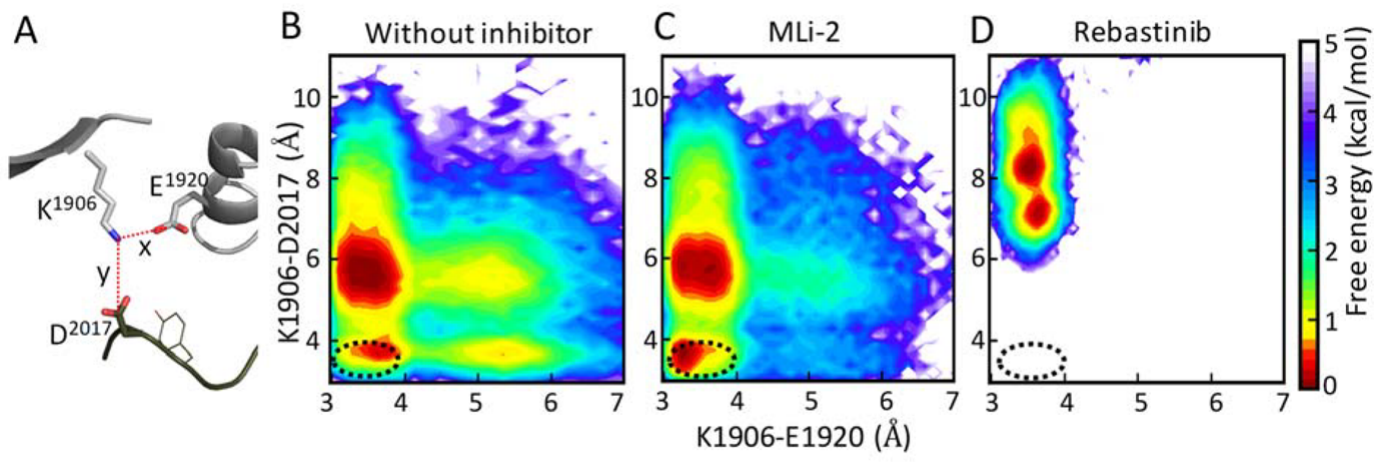
The formation of the regulatory triad. (A) The two distances, Nζ^K1906^–Cδ^E1920^ and Nζ^K1906^–Cγ^D2017^, were measured to characterize the regulatory triad. The two-dimensional free energy profiles projected along two distance coordinates for (B) LRRK2^RCKW^ without inhibitor; (C) Mli-2 bound LRRK2^RCKW^; (D) Rebastinib bound LRRK2^RCKW^. The *x* axis shows the distance of K1906–E1920, and the *y* axis shows the distance of K1906–D2017. The black circle indicates the location where the regulatory triad E1920–K1906–D2017 is formed. Binding of MLi-2 promotes the assembly of a regulatory triad while binding of Rebastinib inhibits it.

The binding of MLi-2 also promoted the assembly of the highly conserved regulatory triad, the salt bridges that define every active kinase (Figure 3A).^31^ The salt bridges between two conserved residues in the N lobe, E1920 in the αC helix and K1906 in β3, and D2017 in the DYG motif in the C lobe are essential for active forms of kinases. The free energy profiles along the distances of the two salt bridges are shown in Figure 4. Compared with the system of RCKW without an inhibitor (Figure 4B), the triad is more stable in the MLi-2-bound system (Figure 4C), which does not form metastable states at a larger K1906–E1920 distance (5–6 Å). This triad also helps to drive the assembly of the R-spine, another hallmark feature of active kinases (Figure 3C, Supporting Information Movie 1).^32^ Consistent with HDX-MD data, the YRD motif, where Y1990 is an R-spine residue, is also stabilized by MLi-2, shown by the smaller RMSD values of residues in the region of the YRD motif (Figure S3). In addition, MLi-2 stabilized residues in the hinge region. Another hallmark signature of an active kinase that is in a fully closed conformation is the position of the hydrophobic residue in the glycine-rich loop, which is F1890 in LRRK2. As seen in Figure 3B, F1890 further stabilizes the hydrophobic bridge between the N and C lobes.

In contrast to MLi-2, Rebastinib occupies the ATP binding site and the adjacent hydrophobic pocket, replacing the space that was occupied by Y2018 of the DYG motif in the MLi-2 structure (Figure 3D, E). This forces the LRRK2 kinase domain into the DYG-Out/BBAminus conformation.^30^ The DYG motif was unable to flip back into the DYG-In orientation for the entire simulation time (Figure S2). Rebastinib breaks the hydrophobic bridge between the N and C lobes, which defines the active kinase; it makes the closing of the active site cleft impossible. When they are uncoupled from the N lobe, the dynamics of the DYG motif and the YRD motif were both also increased compared to the LRRK2^RCKW^ alone, as indicated by the higher RMSD values of residues in both motifs (Figure S3). The isoquinoline of the Rebastinib is positioned against the αC helix and inhibits the movement of the αC helix toward the C lobe, preventing the kinase domain from adopting a closed, active-like conformation. The E1920 on the αC helix is bound to Rebastinib through H-bonding with the two nitrogens on Rebastinib, leaving it far away from D2017 in the DYG motif of the C lobe. Although the salt bridge between K1906 and E1920 was stably present throughout the MD simulations, the regulatory triad never assembled (Figure 4D, Supporting Information Movie 2).

**Figure 5:**
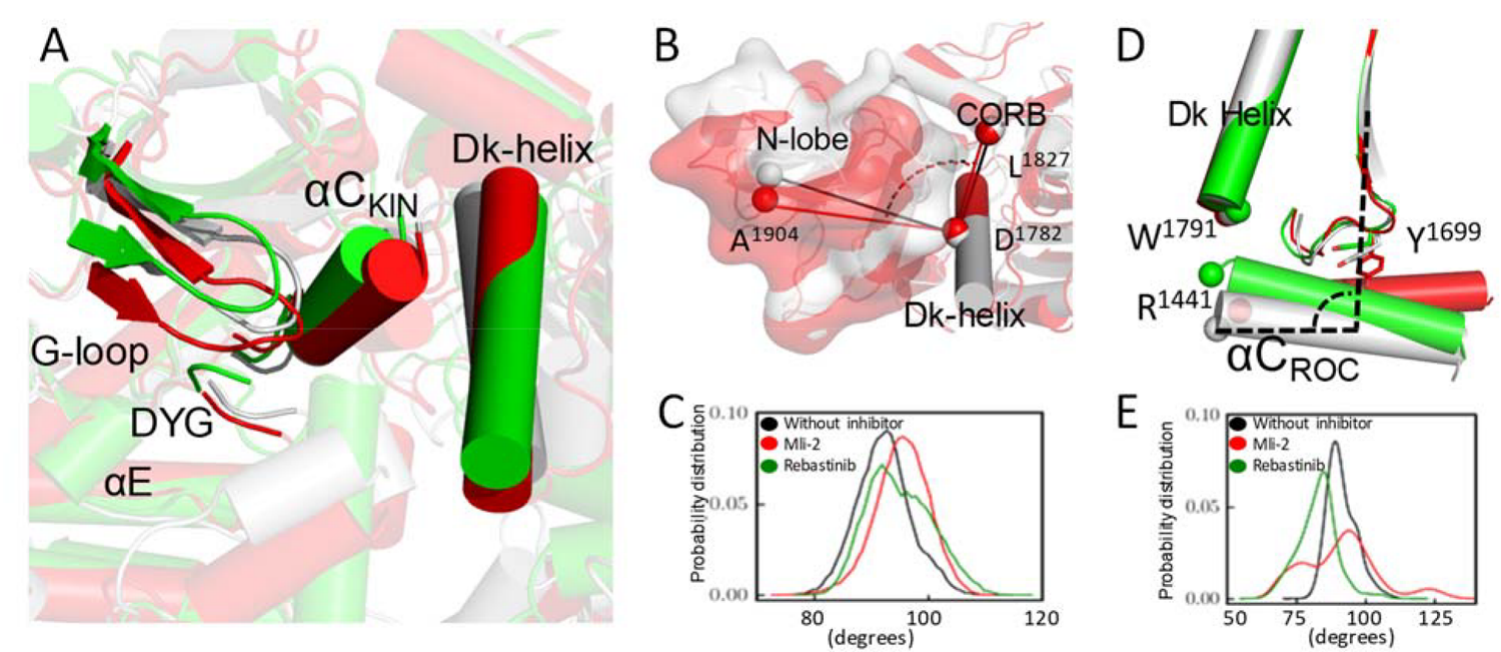
Clustering analysis of the MD conformations. (A) Representative structures from the first cluster of different simulation conditions are colored as follows: RCKW without inhibitor (gray); RCKW/MLi2 (red); RCKW/Rebastinib (green). Structures aligned by the αE and αF helices of the kinase domain show that the G-loop, αC, and Dk helix of RCKW/MLi-2 are closer to the C lobe in the MLi-2-bound structure. In contrast, the RCKW/Rebastinib complex resembles the open conformation of the kinase domain. (B) The representative structures aligned by Dk helix show that when MLi-2 is bound to RCKW, the top of the N lobe moves away from the CORB domain compared to RCKW without an inhibitor. (C) The angle of A1904–D1782–L1827 was measured to show the relative orientation of the N lobe toward the CORB domain. (D) The αC helix in the ROC domain, which hinges at Y1669, tilts toward the C-terminal end of the Dk helix, when Rebastinib is bound. This tilting motion of RCKW/Rebastinib moves R1441 closer to W1791, whereas The R1441 moves way from the W1791 when bound to MLi-2. (E) The angle of R1693–W1434–R1441 was measured to show the tilting of the αC helix.

To further deduce how binding to the kinase inhibitors changes the conformation of LRRK2^RCKW^, we performed clustering analysis to extract representative structures from our simulation data. The first class of each condition, RCKW, RCKW/MLi-2, and RCKW/Rebastinib, is aligned by the stable helices, helixes E and F, in the C lobe of the kinase domain (Figure 5A). While the C lobe aligns well except for the loop regions, the orientations of the N lobe of the kinase domain are different when binding to the two inhibitors. This observation suggests that the N lobe and the C lobe move as independent rigid bodies (Figure S4), while the WD40 domain remains stably anchored to the C lobe in all of the structures solved so far. The Gly-rich loop and the αC helix are closer to the C lobe when LRRK2^RCKW^ binds to MLi-2 compared to the RCKW or RCKW/Rebastinib systems, showing a closed conformation.

We note that the RCKW/MLi-2 structure is still distinctly different from the ATP-bound, fully closed, active conformation, based on PKA,^33^ where the Gly-loop folds over the nucleotide and the αC helix is closer to the β-sheet core of the N lobe (Figure S5). The glycine loop, including the Phe residue at the tip of the G loop, folds over the MLi-2 while the Phe in the ATP:PKI bound structure of PKA (Phe54) folds over onto the DFG+1 residue and reinforces the hydrophobic latch between the N and C lobes. The tip of the αC helix, however, remains anchored to the Dk helix, although it is closer to the activation segment of the kinase domain. When binding to Rebastinib, the N lobe is further away from the C lobe compared to the MLi-2 bound conformation. By occupying the space that was filled by Y2018 in the MLi2-bound structure, Rebastinib essentially breaks the R-spine, which severs the N and C lobes and forces the N-terminus of the αC helix away from the C lobe (Figure 3F). In the inactive full-length LRRK2 structure, the DYG motif forms an inhibitory helix that prevents the assembly of R-spine (Figure S6). This inhibited structure, however, is distinct from the RCKW/Rebastinib structure; it does not correspond to the stable inhibited conformation that is found in the full length LRRK2.

#### Interdomain Communications Across LRRK2 Are Affected Differently by the Two Inhibitors

We then sought to understand how interdomain interactions between the COR-B domain and the kinase domain are involved in regulating the activation of LRRK2. When the kinase domain is stabilized in the closed conformation by MLi-2, the N lobe of RCKW/MLi-2 rotates along the Dk helix of the COR-B domain and moves closer to the COR-B domain (Figure 5B and C). More interactions can be identified between the Dk helix and the COR-B loop than the system without an inhibitor (residues 1721–1725; Figure S7A). In agreement, the COR-B loop also shows a smaller deuterium uptake when LRRK2^RCKW^ binds to MLi-2 (Figure S7B). This orientation resembles the active LRRK2 structure recently solved by Sun’s group.^24^ In contrast to MLi-2, binding of the Rebastinib did not alter the N lobe orientation, and the HDX-MS result also shows no protection effect on the COR-B loop. We also analyzed the reported “seesaw-like” motion of the ROC αC helix relative to the COR-B domain, which was shown to be related to LRRK2 activation.^24^ In the inactive conformation, the C-terminal end of the ROC αC helix stays closer to the C-terminal end of the Dk helix. R1441 at the C-terminal end of the ROC αC helix, in particular, can interact with W1791 at the C terminus of the Dk helix, which anchors the ROC αC helix to the surface of the COR-B domain in the inactive state. Rebastinib binding stabilizes this inactive conformation and tilts the ROC αC helix in the direction toward the Dk helix (Figure 5D, E). On the other hand, binding to MLi-2 stabilizes an active-like conformation, where the C-terminal end of the ROC αC helix moves away from the Dk helix. In this way MLi-2 prevents the interaction between R1441 and W1791, thus disrupting the anchoring of the ROC αC helix to the Dk helix. These changes also most likely promote other interactions with the activation segment of the kinase domain, which is highly dynamic and lies in close proximity to this region (Figure S8).

**Figure 6:**
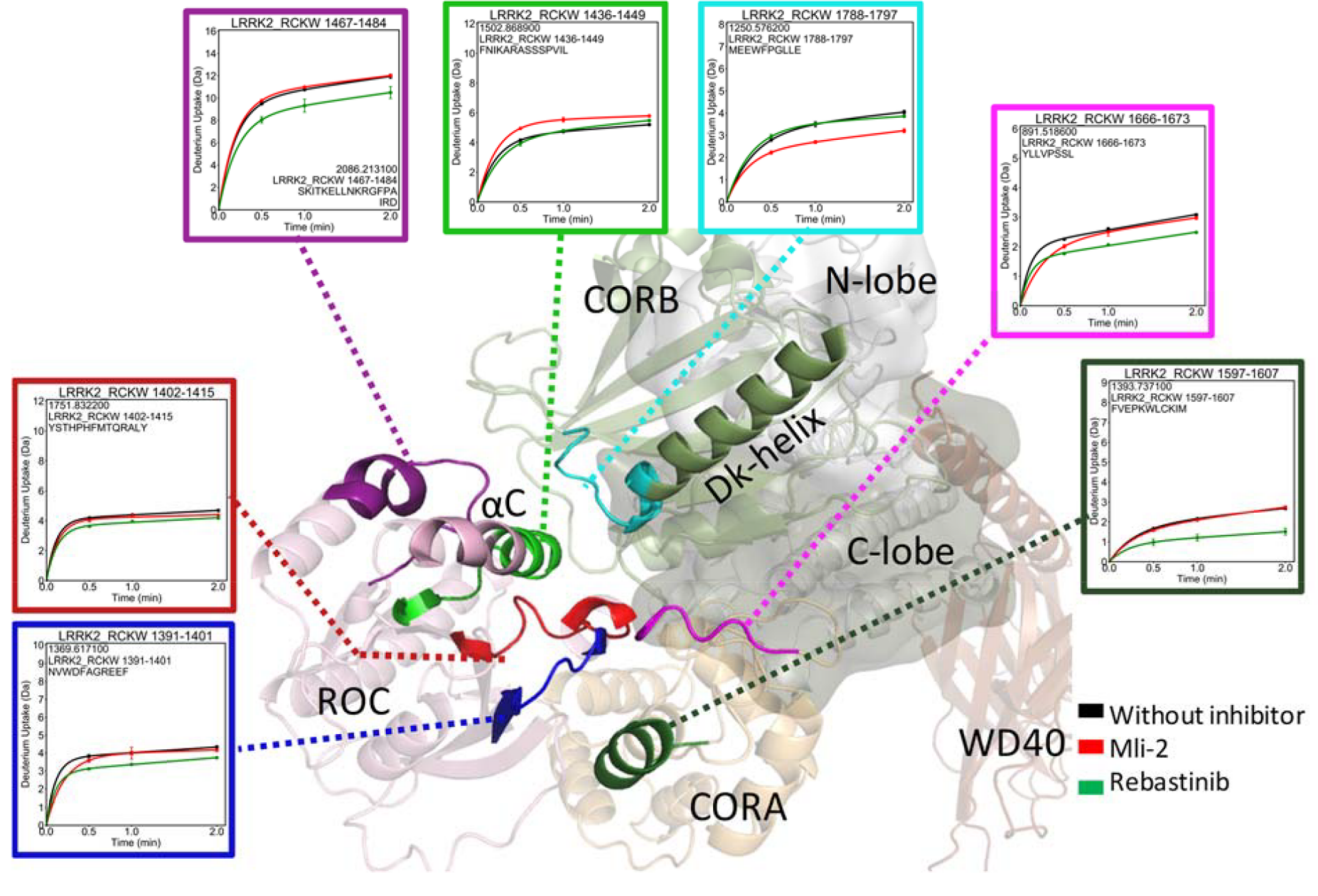
Deuterium uptake of LRRK2 at the ROC, CORA, and CORB interfaces. The deuterium uptake of selected peptides is plotted and mapped on the LRRK2^RCKW^ model. When binding to MLi-2, the ROC αC helix shows increased deuterium uptake while uptake is decreased in the peptide from the Dk helix. For peptides at the domain interfaces, binding of Rebastinib globally reduces their deuterium uptake while MLi-2 binding has no effect.

The dynamic changes related to the domains that flank the kinase domain were also captured by HDX-MS (Figure 6 and Table S1). The peptide that covers the C-terminus of the ROC αC helix (residues 1436–1449) shows increased deuterium uptake in the presence of MLi-2. This is consistent with the MD results showing that the interaction between W1791 and R1441 was disrupted by the binding of MLi-2, releasing the ROC αC helix and making it more dynamic and solvent accessible. Interestingly, two PD mutations are included in this important peptide, R1441 and N1437. In addition, the decreased deuterium uptake of the C terminus of the Dk helix (residues 1788–1795) can be attributed to its increased interactions with the activation segment observed in the simulation that shielded these residues from the solvent (Figure S8). Binding of the Rebastinib does not show any effect on the deuterium uptake of either the ROC αC helix or the Dk helix. However, peptides that cover the residues at the interface of ROC–CORA (residues 1391–1401), CORA–CORB (residues 1666–1673), and ROC–CORB (residues 1467–1484) show decreased deuterium uptake in the presence of Rebastinib, while binding to MLi-2 has no protection effect. This suggests that the anchoring of the ROC αC helix to the Dk helix not only influences the communication that takes place between the kinase domain and the CORB domain but also alters the cross-talk within the ROC, CORA, and CORB domains.

#### Type I and Type II Inhibitors Shift the Energy Landscapes for the LRRK2 Conformational Space Differently

**Figure 7:**
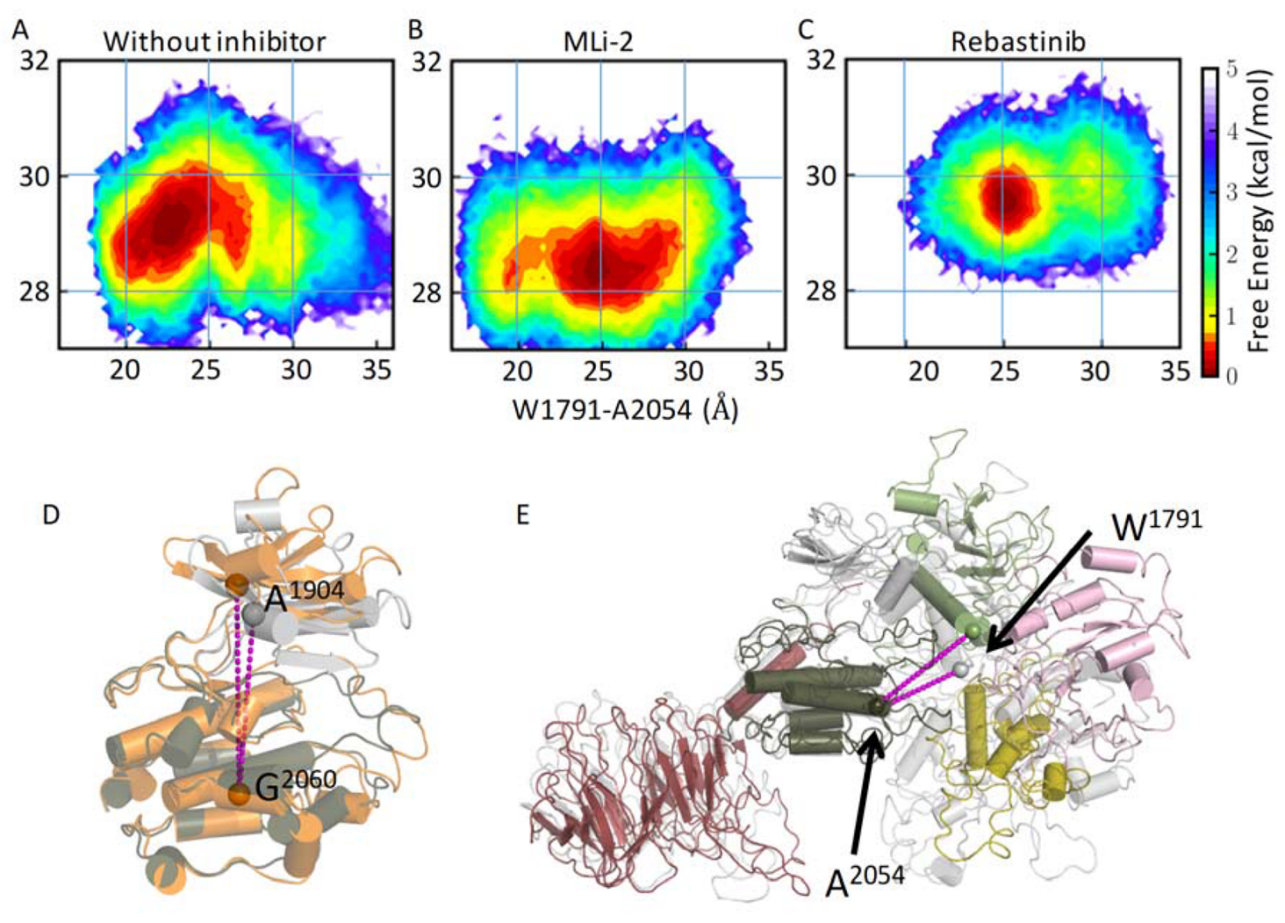
Binding of the inhibitors alters LRRK2^RCKW^ dynamics. The conformational free-energy landscapes of RCKW without an inhibitor (A), RCKW/MLi2 (B), and RCKW/Rebastinib (C). (D) The open (orange) and closed (gray) conformations of the kinase domain are characterized by the distance from the N lobe (Cα^A1904^) to the C lobe (Cα^G2060^). (E) The extended (colored) and compact (gray) conformations of LRRK2^RCKW^ are characterized by the distance between CORB (Cα^W1791^) and C lobe (Cα^A2054^). Binding of MLi-2 stabilizes the kinase domain in a closed/compact state. In contrast, the Rebastinib-bound LRRK2^RCKW^ is trapped in an open/extended state and is less dynamic.

Using GaMD simulations, we had previously captured the breathing dynamics of LRRK2^RCKW^ where the ROC–CORA, CORB, N-lobe- and C-lobe-WD40 domain move as rigid bodies^28^ (Figures 5 and S4). To explore how binding of MLi-2 and Rebastinib affects the breathing dynamics of LRRK2^RCKW^ as well as the kinase domain equilibrium, we computed a two-dimensional energy landscape for each simulation condition (Figure 7A–C). The open and closed conformations of the kinase domain were measured by the relative position of the N and C lobes, which are represented by Cα^A1904^ and Cα^A2060^. The distance change correlates with the transition between the inactive conformation where the N and C lobes are uncoupled and the active-like conformation of the kinase domain where the two lobes come together (Figure 7D). For all of the simulation conditions, the kinase domain of LRRK2^RCKW^ toggles between open and closed conformations (*y* axis of Figure 7A–C). The MLi-2-bound LRRK2^RCKW^ has an energy minimum at 28.5 Å, smaller than the minimum positions of the LRRK2^RCKW^ without an inhibitor (29.2 Å) and Rebastinib-bound LRRK2 ^RCKW^ (29.8 Å).

The distance between the C lobe and the CORB domain (the distance between Cα^A2054^ and Cα^W1791^ in Figure 7E) was measured to demonstrate the large-scale breathing motion. Both the extended and compact conformations of LRRK2^RCKW^ were sampled according to the distributions along the *x* axis of Figure 7A–C. MLi-2-bound LRRK2 exhibits two local energy minima, one at 20 Å, representing a more compact configuration than that at the RCKW without an inhibitor minimum (23 Å). The distribution along the *x* axis for Rebastinib-bound LRRK2 shifts to a more extended configuration and has a minimum at 25 Å. We also calculated the 2D energy profiles using the C lobe–CORA distance and the kinase conformational coordinate in Figure S9. Similarly, only MLi-2-bound LRRK2^RCKW^ has a population representing a very compact LRRK2, while the Rebastinib-bound LRRK2^RCKW^ shows minimal population for the compact configuration.

**Figure 8:**
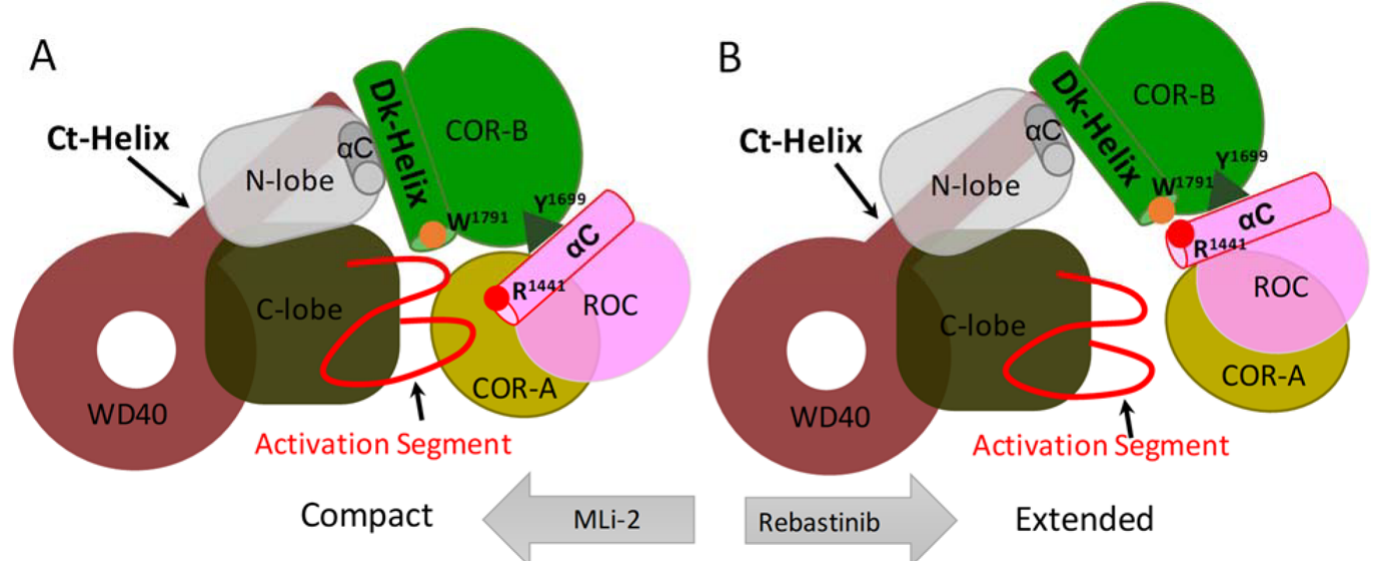
Cartoon representation of the compact and extended states of LRRK2^RCKW^. (A) The kinase domain is stabilized in a closed conformation when the LRRK2^RCKW^ is in a compact conformation, where the ROC, CORA, and CORB domains are closer to the C lobe of the kinase domain. In this closed conformation, the αC helix in the ROC domain tilts away from the Dk helix. (B) The open, inactive conformation of the kinase domain promotes the extended conformation of LRRK2^RCKW^. In the extended conformation, the αC helix in the ROC domain is closer to W1295 in the Dk helix of the CORB domain.

Based on the three energy profiles, the closed or open conformation of the kinase domain is in concert with the compact or extended conformation of LRRK2^RCKW^, respectively, illustrating the correlation between the dynamics of the kinase domain and the breathing dynamics of LRRK2^RCKW^. Binding of MLi-2 shifts the kinase domain into a closed/compact state and disrupts the coherent movement between the closed/compact and open/extended states (Figure 7B). MLi-2 bound LRRK2^RCKW^ was able to sample smaller distance distribution between the C lobe and COR-B. On the other hand, binding of Rebastinib locks the kinase domain in an open, inactive state, which corresponds to the extended state, and the compact RCKW conformation is rarely sampled (Figures 7C and S9).

## Discussion

The function of LRRK2 is mediated by two finely tuned regulatory switches, a kinase domain and a GTPase (ROC) domain, which regulate how LRRK2 toggles between its active and inactive states.^28,34−36^ Previous studies showed that different distributions of LRRK2 in the cell and different phenotypes are captured in the presence of type I or type II kinase inhibitors.^17,18^ The study of Rab protein phosphorylation with type I and type II inhibitors also indicates that the function of LRRK2 is more than the activity of the kinase domain. In this study, we used GaMD simulations coupled with HDX-MS to analyze the dynamic changes of LRRK2 and its interdomain allosteric communications. We have shown that the kinase domain serves as a central hub for interdomain communication and is the major driver for LRRK2’s conformational transitions.^27,37^ We also identified the key interactions within and between domains, which connect different effects of kinase inhibitors to the overall conformational states of LRRK2.

Here, we use MD simulations to show how different inhibitors capture different conformational ensembles and then validated these conformational differences using HDX-MS. Even though MLi-2 (type I) and Rebastinib (type II) both inhibit the kinase activity with high affinity, they drive LRRK2 into different conformational states. Binding of MLi-2 reduced the solvent accessibility of the kinase domain not only in the active site cleft where the MLi-2 is directly docked but also in regions in the C lobe that lie far from the active site cleft but nevertheless interact with other domains. The HDX-MS data indicate that binding of Rebastinib traps the kinase domain in an open conformation, which separates the C lobe of the kinase domain from the N lobe, making it more solvent exposed and more dynamic. The MD simulation results show that Rebastinib-bound LRRK2 results in a more dynamic kinase domain that favors an open, inactive conformation where the N and C lobes are uncoupled. In contrast, the MLi-2-bound LRRK2 simulations show that the closed, active-like conformations are sampled more frequently.

The changes in conformation and dynamics that are caused by binding of kinase inhibitors also extend beyond the kinase domain in LRRK2^RCKW^. In this study, we showed the distinct roles of the Dk helix in the CORB domain, which is anchored to the αC helix in the N lobe of the kinase domain. This Dk helix is part of the allosteric cross-talk that takes place between the kinase and ROC domains. Our results are in agreement with the recent report showing that bridging of the ROC domain and the activation segment in the kinase domain is different in the active and inactive structures.^24^ When the kinase is locked in an open conformation by Rebastinib, the communication between the N and C lobes of the kinase domain is disrupted, which is consistent with the N lobe functioning as an independent rigid body.^23^ The C lobe of the kinase becomes more dynamic and uncoupled from the CORB domain.

The interactions between the CORB–CORA, CORB–ROC, and the CORA–ROC domains are more stable based on the reduced solvent accessibility of the peptides that are located at these interfaces. LRRK2^RCKW^ is stabilized in an extended state by Rebastinib, whereas binding of MLi-2 stabilizes a closed, active-like conformation of the kinase domain (Figure 8). With MLi-2, the overall dynamic features of the kinase domain are reduced, and the disordered regions surrounding the activation segment become more ordered, and as a consequence, more extensive interactions can be identified between the Dk helix and the kinase domain.

Our results suggest that different kinase inhibitors can be used to trap full-length LRRK2 in specific states for future functional studies. The assembly of LRRK2 on MT is promoted by the type I kinase inhibitors, such as MLi-2.^38−41^ We show with MD simulations how a very compact global configuration (Figure 7B) is captured in the MLi-2 bound structure, and this correlated well with the enhanced global protection seen with our HDX-MS data. This compact state can fit into the cryo-EM density of the helical assembly on MT.^17^ The Rebastinib bound LRRK2 simulations reveal an extended conformation (Figure 7C) that provides the molecular basis for the inhibition of LRRK2 filament formation observed in experiments.^17,18^ These observations are significant considering the starting structure for the simulations is a monomeric LRRK2^RCKW^ with an inactive kinase domain. Moreover, the metastable states responsible for the allosteric regulation in the kinase and flanking domains can serve as a starting point for identifying new therapeutic targets for treating PD—*in silico* ensemble screening and docking^42^ can now be used to target these interfaces and the transient dynamic states highlighted by our simulations.

## Material and Method

### Hydrogen–Deuterium Exchange Mass Spectrometry

LRRK2^RCKW^ protein (residue 1327 to 2527) was expressed and purified from Sf9 cells as described previously.^18^ The protein used for hydrogen/deuterium exchange mass spectrometry (HDX-MS) was analyzed by SDS-PAGE (Figure S10). HDX-MS was performed using a Waters Synapt G2Si equipped with a nanoACQUITY UPLC system with H/DX technology and a LEAP autosampler. The LRRK2^RCKW^ concentration was 5 μM in LRRK2 buffer containing 20 mM HEPES/NaOH pH 7.4, 800 mM NaCl, 0.5 mM TCEP, 5% glycerol, 2.5 mM MgCl_2_, and 20 μM GDP. MLi-2 and Rebastinib were dissolved in DMSO to make a 10 mM stock solution. Inhibitors were added into LRRK2^RCKW^ protein in LRRK2 buffer to make the LRRK2/inhibitor complex samples. The deuterium uptake was measured in LRRK2 buffer in the presence and absence of the kinase inhibitor MLi-2 (50 μM) or Rebastinib (50 μM). For each deuteration time, 4 μL of the complex was equilibrated to 25 °C for 5 min and then mixed with 56 μL of D_2_O LRRK2 buffer for 0, 0.5, 1, or 2 min. The exchange was quenched with an equal volume of quench solution (3 M guanidine, 0.1% formic acid, pH 2.66). The quenched sample (50 μL) was injected into the sample loop, followed by digestion on an in-line pepsin column (immobilized pepsin, Pierce, Inc.) at 15 °C. The resulting peptides were captured on a BEH C18 Vanguard precolumn, separated by analytical chromatography (Acquity UPLC BEH C18, 1.7 μM, 1.0 × 50 mm, Waters Corporation) using a 7–85% acetonitrile gradient in 0.1% formic acid over 7.5 min and electrosprayed into the Waters SYNAPT G2Si quadrupole time-of-flight mass spectrometer. The mass spectrometer was set to collect data in the Mobility, ESI+ mode; mass acquisition range of 200–2000 (*m*/*z*); scan time, 0.4 s. Continuous lock mass correction was accomplished with infusion of leu-enkephalin (*m*/*z* = 556.277) every 30 s (mass accuracy of 1 ppm for the calibration standard). For peptide identification, the mass spectrometer was set to collect data in MS^E^, ESI+ mode instead.

The peptides were identified from triplicate MS^E^ analyses of 10 μM LRRK2^RCKW^, and data were analyzed using PLGS 3.0 (Waters Corporation). Peptide masses were identified using a minimum number of 250 ion counts for low energy peptides and 50 ion counts for their fragment ions. The peptides identified in PLGS were then analyzed in DynamX 3.0 (Waters Corporation) using a cutoff score of 6.5 and error tolerance of 5 ppm and requiring that the peptide be present in at least two of the three identification runs. The peptides reported on the coverage maps are those from which data were obtained. The relative deuterium uptake for each peptide was calculated by comparing the centroids of the mass envelopes of the deuterated samples vs the undeuterated controls.^43^ For all HDX-MS data, at least two replicates of protein ID runs were applied to check the quality between protein batches. Each time point was analyzed with three replicates. Data are represented as mean values ± SEM of three replicates. The deuterium uptake was corrected for back-exchange using a global back exchange correction factor (typically 25%) determined from the average percent exchange measured in disordered termini of various proteins.^44^ Deuterium uptake plots were generated in DECA (github.com/komiveslab/DECA), and the data are fitted with an exponential curve for ease of viewing.^45^

### Gaussian Accelerated Molecular Dynamics (GaMD) Simulation

The LRRK2^RCKW^ model for simulations was prepared based on the reported LRRK2^RCKW^ structure (PDB: 6VP6). The inhibitor bound structures were modeled based on PDB: 5OPB (MLi-2) and PDB: 6MWE (Rebastinib). Modeller was used to model the missing loops.^46^ The Protein Preparation Wizard was used to build missing side chains and model charge states of ionizable residues at neutral pH. Hydrogens and counterions were added, and the models were solvated in a cubic box of TIP4P-EW water molecules^47^ and 150 mM KCl with a 10 Å buffer in AMBER tools. AMBER16 was used for energy minimization, heating, and equilibration steps, using the CPU code for minimization and heating and GPU code for equilibration. Parameters from the Bryce AMBER parameter database were used for phosphoserine and phosphothreonine.^48^ Systems were minimized by 1000 steps of hydrogen-only minimization, 2000 steps of solvent minimization, 2000 steps of ligand minimization, 2000 steps of side-chain minimization, and 5000 steps of all-atom minimization. Systems were heated from 0 to 300 K linearly over 200 ps with 2 fs time-steps and 10.0 kcal/mol/Å position restraints on protein. Temperature was maintained with a Langevin thermostat. Constant pressure equilibration with an 8 Å nonbonded cutoff and particle mesh Ewald was performed for 300 ps with protein and peptide restraints, followed by 900 ps of unrestrained equilibration. Gaussian accelerated MD (GaMD) was used on GPU-enabled AMBER16 for enhanced conformational sampling.^49^ GaMD applies a Gaussian distributed boost energy to the potential energy surface to accelerate transitions between metastable states while allowing accurate reweighting. Both dihedral and total potential boosts were used simultaneously. Potential statistics were collected for 2 ns standard MD followed by 2 ns of GaMD, during which boost parameters were updated. Each GaMD simulation was equilibrated for 10 ns. For each construct, 10 independent replicates of 210 ns production GaMD were run in the NVT ensemble, for an aggregate of 2.1 μs of accelerated MD.

Free energy landscapes were projected along selected conformational coordinates (e.g., Figure 4B–D, and Figure 7A–C). For a 2D space of interest, we constructed a 2D histogram with a total number of *M* bins. The weighted histogram at bin *m* can be determined by
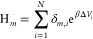
where Δ*V*_*i*_ is the boost potential at the *i*th frame, *N* is the total number of frames, and δ_*m*,*i*_ is an indicator function that determines if frame *i* falls into bin *m*. The Maclaurin series expansion methodwas used to approximate the exponential term.^50^ The free energy profile can be determined by
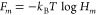
The “measure cluster” command in VMD was used to perform clustering analysis. The total number of clusters was 20. Molecular structures were rendered using PyMol.

## Supporting Information Available

Figure S1, the HDX-MS peptides of LRRK2^RCKW^ without inhibitor, with MLi-2, and with Rebastinib at 2 min; Figure S2, analysis of the frequency of the DYG-in and DYG-out conformations of the kinase domain in LRRK2^RCKW^; Figure S3, the RMSD distribution of selected regions in the kinase domain; Figure S4, representative structures from MD clustering analysis showing the N lobe of the kinase domain; Figure S5, alignment between PKA and the representative structure of LRRK2/MLi-2; Figure S6, the inactive full-length LRRK2 with a broken regulatory spine; Figure S7, comparison of the orientation of the N lobe relative to COR-B domain in different conditions; Figure S8, analysis of the frequency of interactions between the kinase domain and CORB domain; Figure S9, two dimensional (2D) free energy profiles showing the coupling between the conformation of the kinase domain and the overall conformation of LRRK2^RCKW^; Figure S10, SDS PAGE analysis of LRRK2^RCKW^ protein purification from S9 cells (PDF)
The table of HDX-MS data for the deuterium uptake of peptides (XLSX)
Simulation movie of MLi-2 bound LRRK2 kinase domain (AVI)
Simulation movie of Rebastinib bound LRRK2 kinase domain (AVI)

## Supplementary Material

cb2c00868_si_002.xlsx

cb2c00868_si_003.avi

cb2c00868_si_004.avi
