# Supplementary material for: Capturing Differences in the Regulation of LRRK2 Dynamics and Conformational States by Small Molecule Kinase Inhibitors
Source: ACS Chem Biol. 2023 Apr 12;18(4):810–21. doi: 10.1021/acschembio.2c00868 (PMC10127209; doi:10.1021/acschembio.2c00868)
Supplement: Supplementary file 1 — cb2c00868_si_001.pdf [file cb2c00868_si_001.pdf]

Capturing differences in the regulation of LRRK2 dynamics and conformational states by small molecule kinase inhibitors.

Jui-Hung Weng[1¶], Wen Ma[2¶*], Jian Wu[1], Pallavi Kaila Sharma[1], Steve Silletti[2], J. Andrew McCammon[1,2], Susan Taylor[1,2,*]

[1] Department of Pharmacology, University of California, San Diego, CA 92093, USA

[2] Department of Chemistry and Biochemistry, University of California, San Diego, CA 92093, USA

¶These authors contributed equally to this work
* corresponding authors WM and SST.

Email: staylor@ucsd.edu; w1ma@ucsd.edu

Supplementary Figure 1

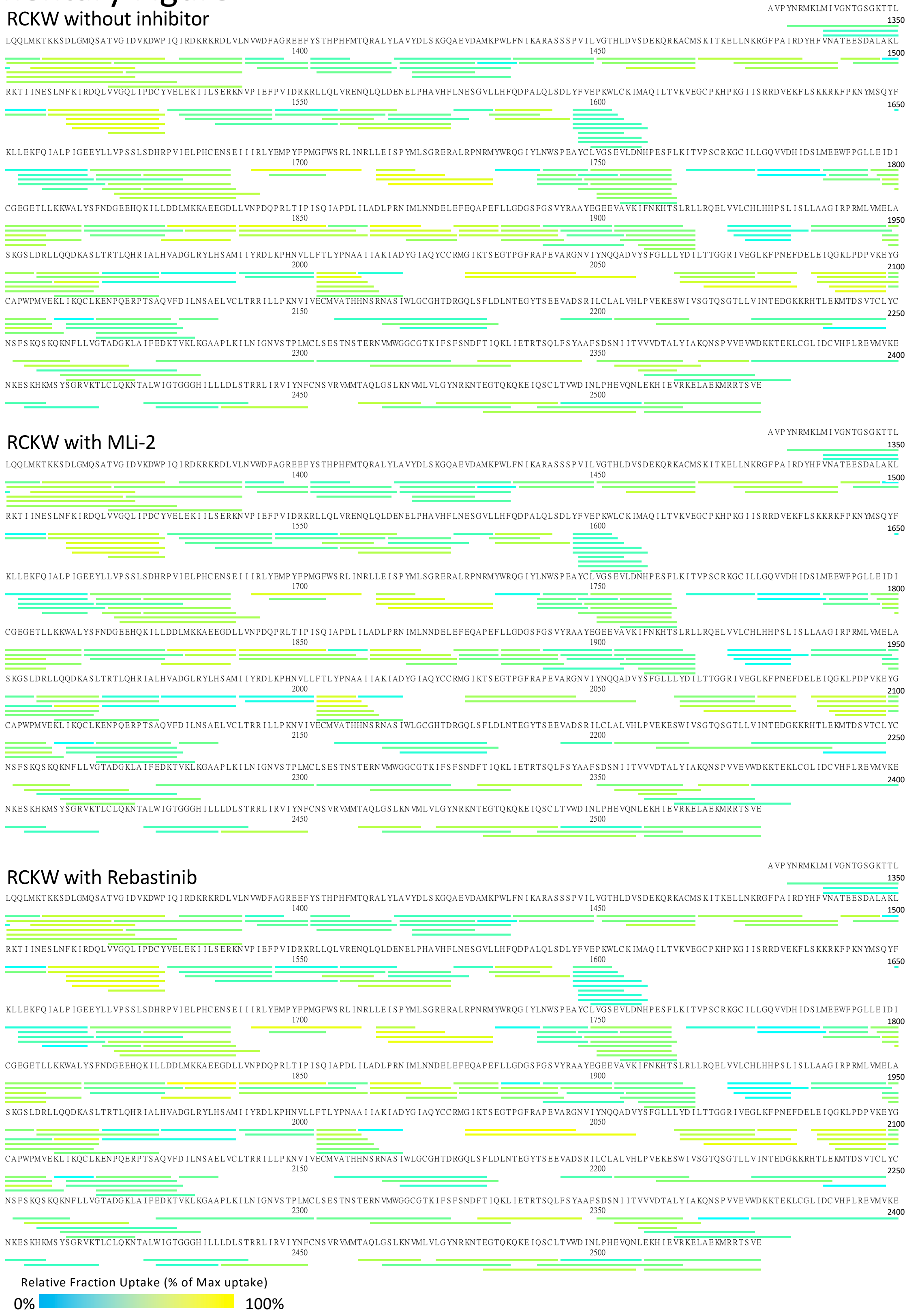

Figure S1. The identified HDX-MS peptides of LRRK2^{RCKW}. The graphs show the relative deuterium exchange of peptides from the N to C terminus of LRRK2 at 2 minutes. Each colored line represents a peptide identified in the MS under all three conditions, with a coverage of 89.9% and redundancy of 2.98. In each panel, the peptides are colored based on their relative fractional uptake under different conditions, from top to bottom: RCKW without inhibitor, RCKW with MLi-2, and RCKW with Rebastinib.

Supplementary Figure 2

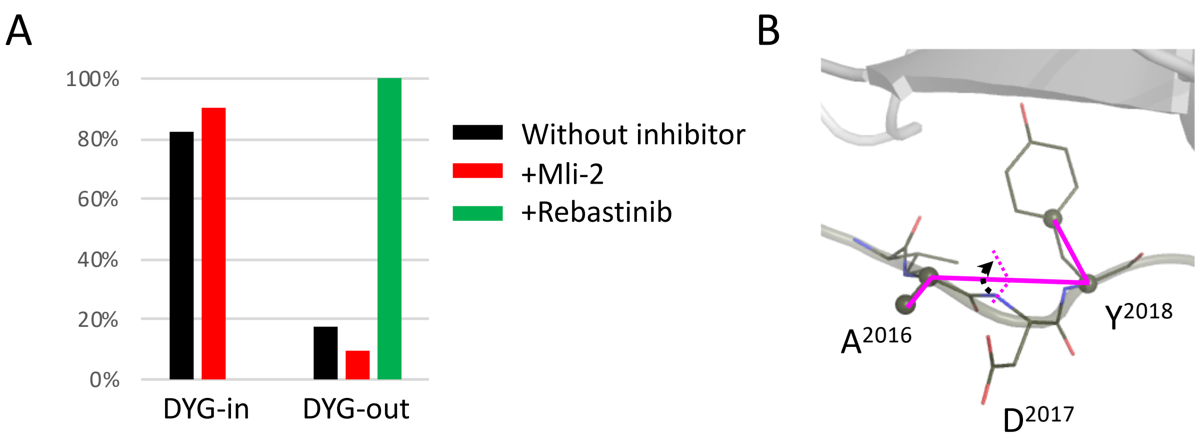

Figure S2. The conformations of the DYG motif. (A) The frequency of DYG-in and DYG-out conformations. Binding of Mli-2 promotes the DYG-in conformation and binding of Rebastinib locks the DYG motif in a DYG-out conformation. (B) The DYG-in or DYG-out orientation is measured by the dihedral angle of O^{A2016}-$C\alpha^{A2016}$-$C\alpha^{Y2018}$-$C\gamma^{Y2018}$.

Supplementary Figure 3

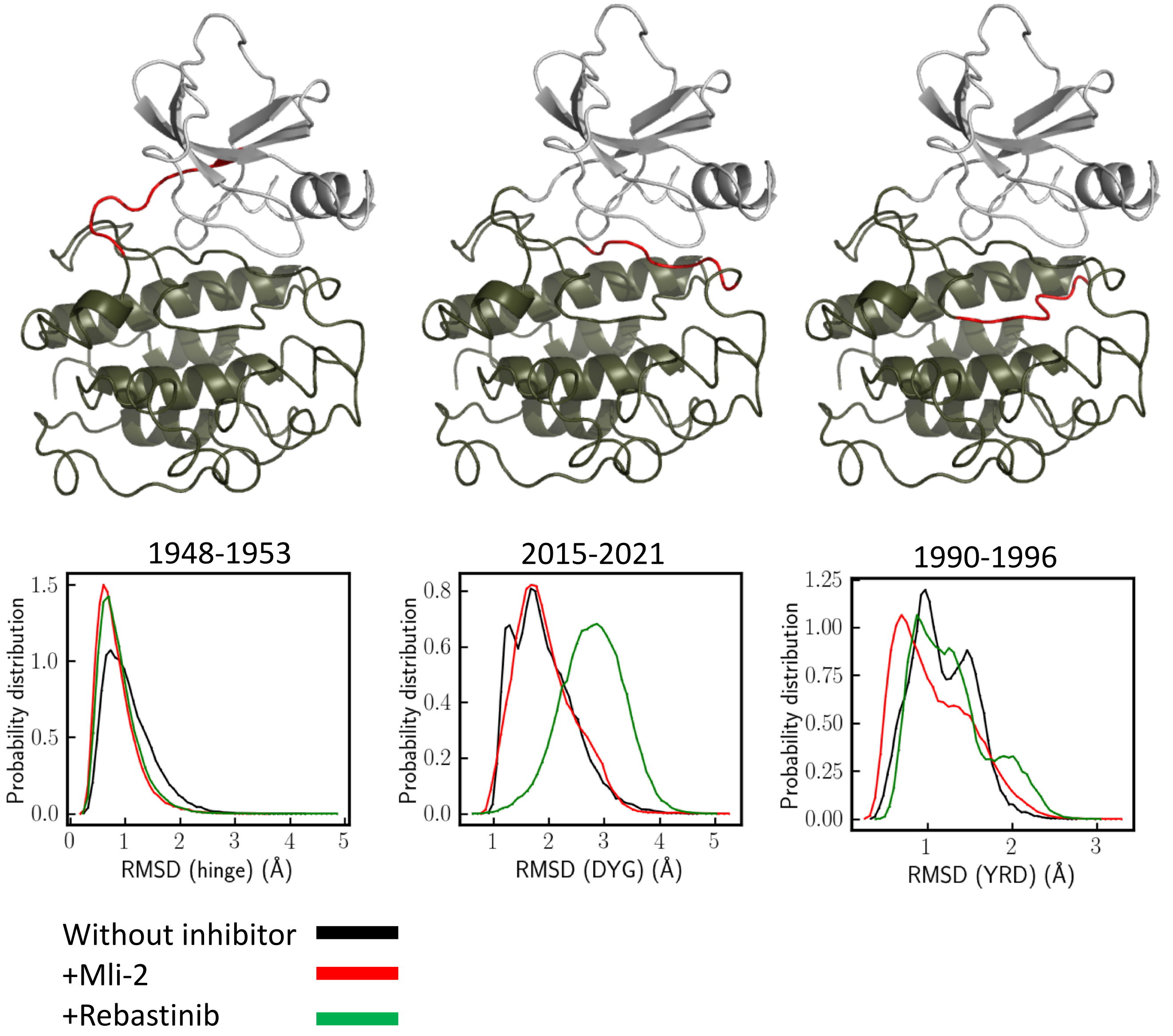

Figure S3. The dynamics of the kinase domain. The RMSD distributions of selected regions (indicated in red): hinge (residues 1948-1953), DYG motif (residues 2015-2021) and YRD motif (residues 1990-1995).

Supplementary Figure 4

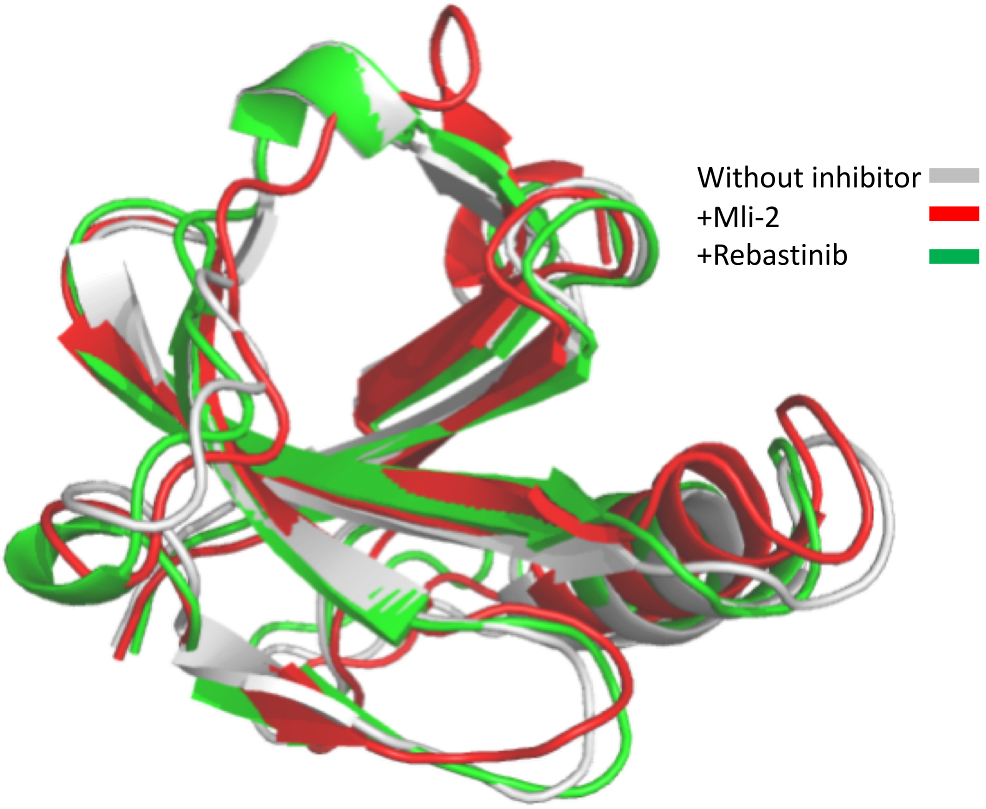

Figure S4. The kinase N-lobe moves as a rigid body in the MD simulations. (A) After clustering analysis on the MD conformations under each condition, a representative structure from the first cluster is displayed (RCKW without inhibitor in gray; RCKW/MLi2 in red; RCKW/Rebastinib in green). When the N-lobe is aligned using the beta sheets, only minor changes are observed, with small variations in the Gly-loop and the αC helix. The N-lobe moves as a rigid body throughout the simulations.

Supplementary Figure 5

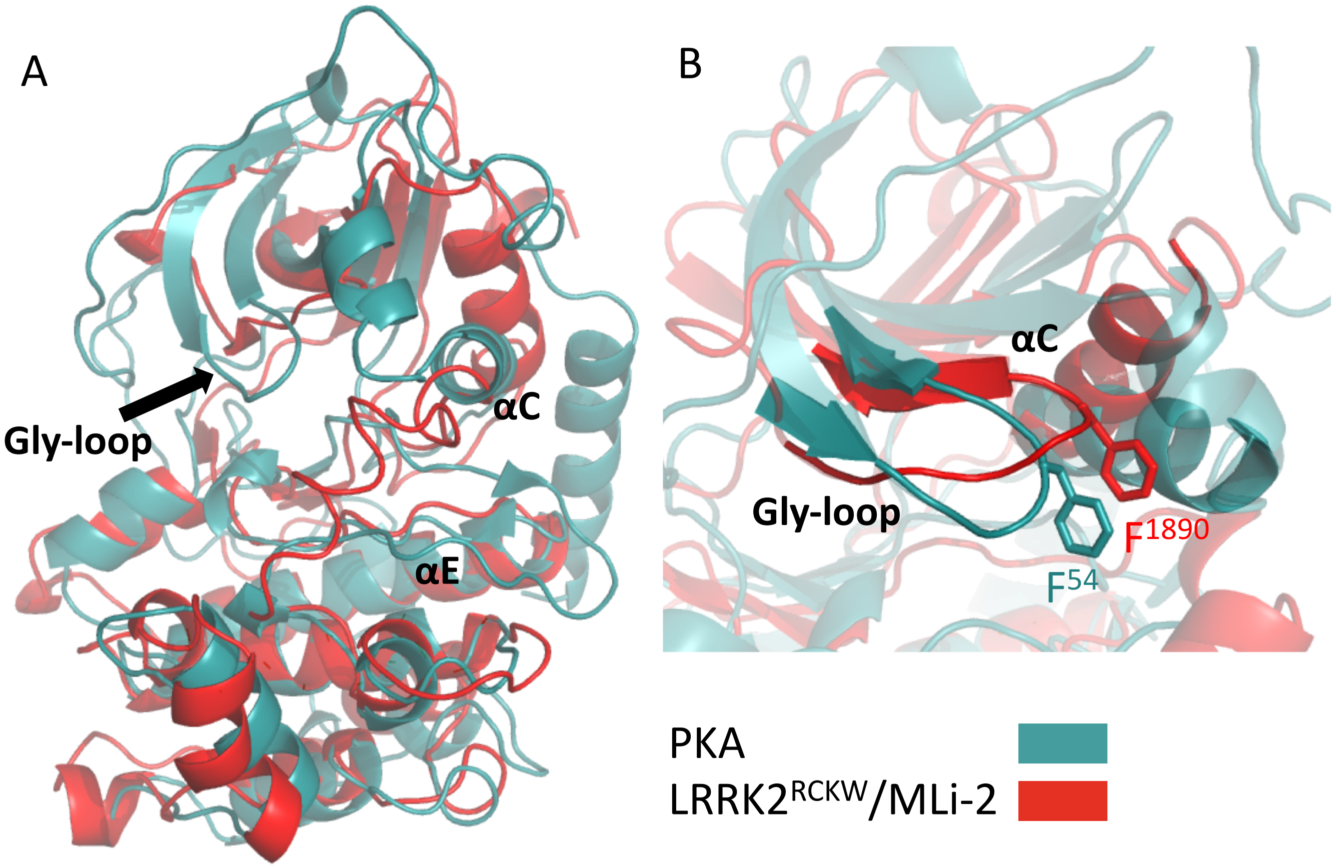

Figure S5. Aligning PKA to the representative conformation of LRRK2/MLi-2. (A) The kinase domain of the representative structure of LRRK2^{RCKW}/MLi-2 from the clustering analysis (red) is aligned to the PKA catalytic subunit (pdb: 1atp) shown in cyan. (B) Zoom-in view of the N-lobe of the kinase domain.

Supplementary Figure 6

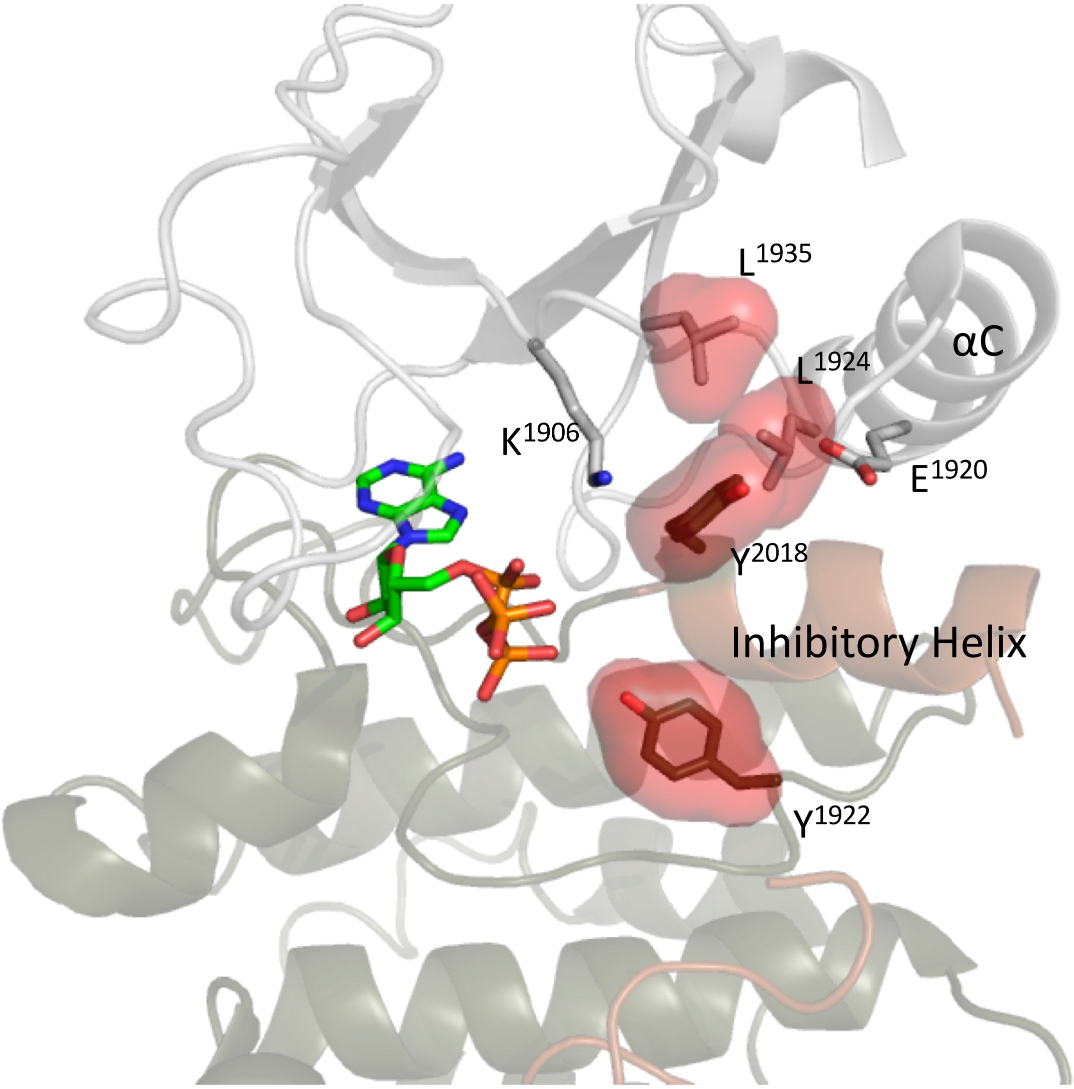

Figure S6. The regulatory spine of the Full-length LRRK2. The R-spine is broken in the inactive full-length LRRK2 (pdb:7lhw). The inhibitory helix of the DFG motif prevents the assembly of R-spine.

Supplementary Figure 7

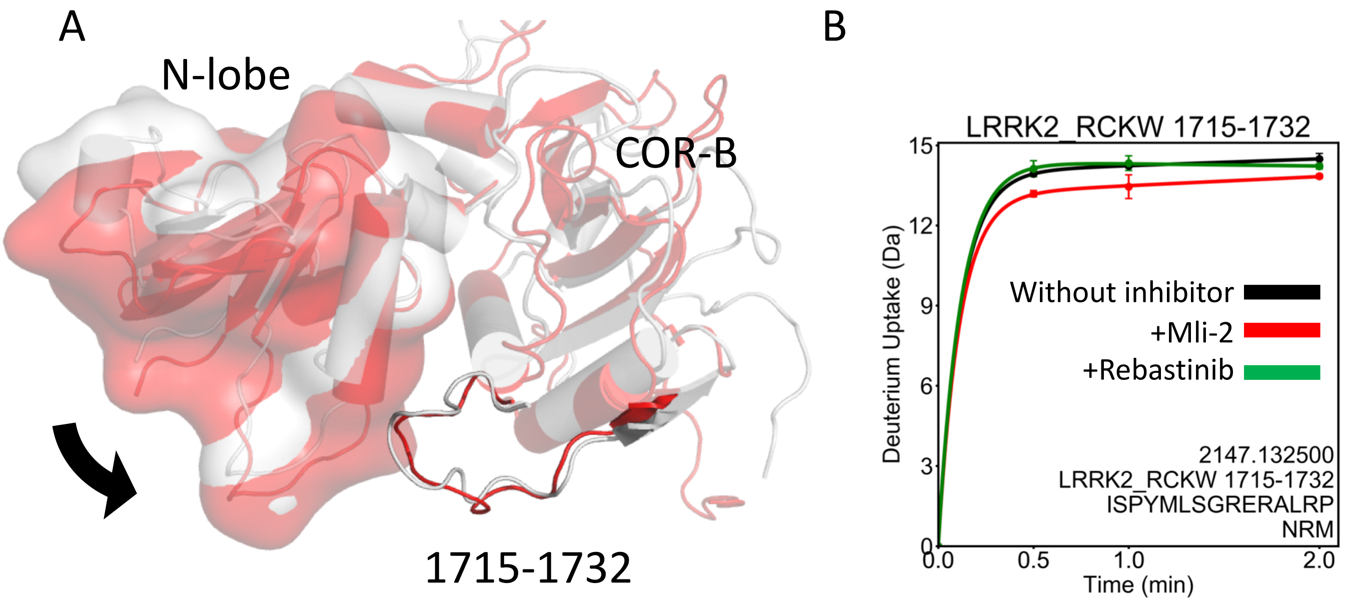

Figure S7. Comparing the orientation of the N-lobe relative to COR-B domain in different conditions. (A) For the MLi-2 bound LRRK2^{RCKW} (shown in red), the N-lobe of the kinase domain moves closer to the COR-B loop (residues 1715-1732), compared to the LRRK2^{RCKW} without an inhibitor (shown in gray). (B) The deuterium uptake of peptide 1715-1732 is reduced in the presence of MLi-2.

Supplementary Figure 8

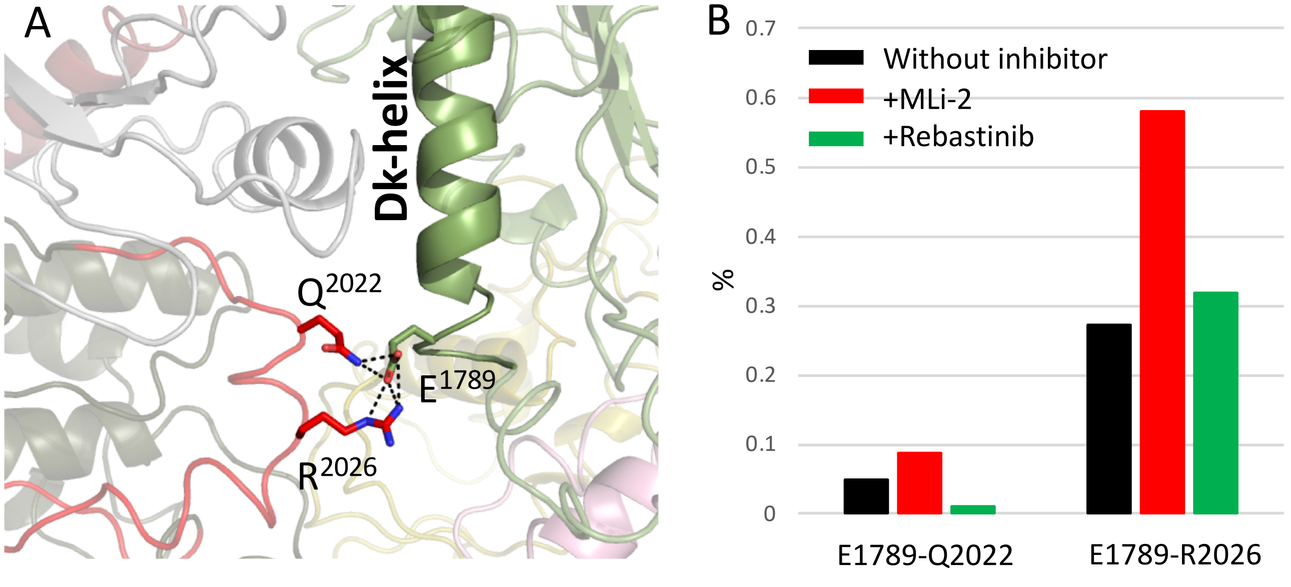

Figure S8. Interactions between the kinase domain and the CORB domain. (A) Interactions between the Activation Segment (Q2022 and R2026) and Dk-helix (E1789) captured in the simulation. (B) Binding of MLi-2 promotes the interactions between E1789-Q2022 and between E1789-R2026.

Supplementary Figure 9

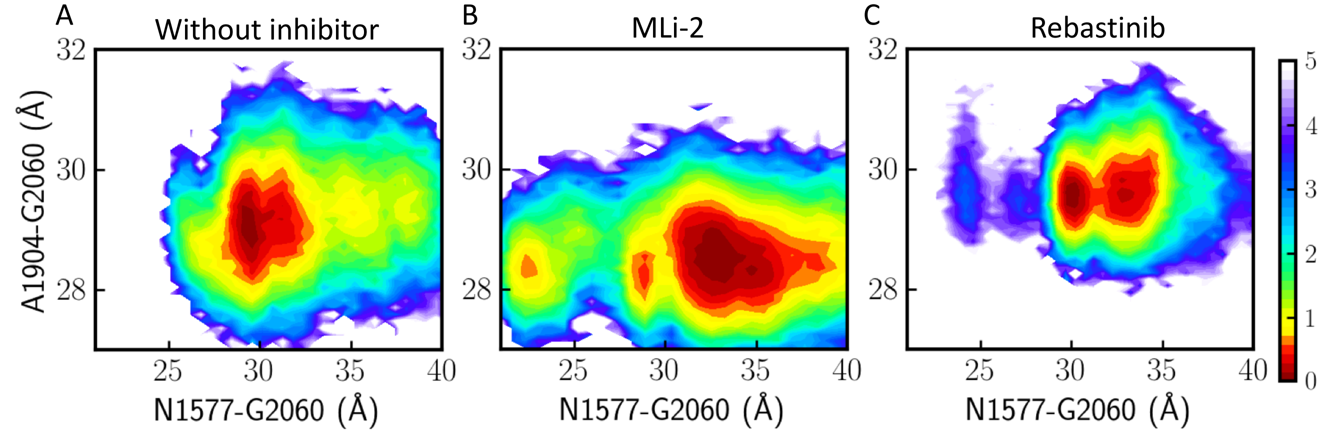

Figure S9. Two dimensional free energy profiles projected along two distance coordinates under different conditions (A) LRRK2^{RCKW} without inhibitor; (B) Mli-2 bound LRRK2^{RCKW}; (C) Rebastinib bound LRRK2^{RCKW}. The x-axis measures the distance between the kinase C-lobe (G2060) and the COR-A domain (N1577). The y-axis measures the distance between the kinase C-lobe (G2060) and kinase N-lobe (A1904).

Supplementary Figure 10

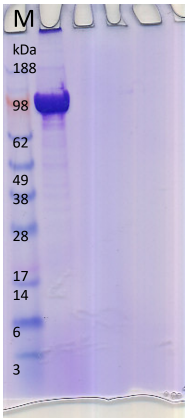

Figure S10. The Representative SDS gel of LRRK2^{RCKW} purification from S9 cells. 5 µg of purified LRRK2^{RCKW} protein is mixed with SDS sample buffer containing 200 mM DTT and loaded onto the gel. The gel shows the protein is in good quality. The gel also includes a molecular weight marker (M).
